# Using fecal microbiota as biomarkers for predictions of performance in the selective breeding process of pedigree broiler breeders

**DOI:** 10.1371/journal.pone.0216080

**Published:** 2019-05-07

**Authors:** Sandra Díaz-Sánchez, Allison R. Perrotta, Isaac Rockafellow, Eric J. Alm, Ron Okimoto, Rachel Hawken, Irene Hanning

**Affiliations:** 1 Department of Food Science, University of Tennessee, Knoxville, TN, United States of America; 2 SaBio IREC (CSIC-UCLM-JCCM), Ciudad Real, Spain; 3 Department of Civil & Environmental Engineering, MIT, Cambridge, MA, United States of America; 4 Department of Biological Engineering, MIT, Cambridge, MA, United States of America; 5 The Center for Microbiome Informatics and Therapeutics, MIT, Cambridge, MA, United States of America; 6 Cobb-Vantress, Inc. Research and Development, Genomics and Quantitative Genetics, Siloam Springs, AR, United States of America; 7 The Graduate School of Genome Sciences and Technology, University of Tennessee, Knoxville, TN, United States of America; University of Illinois, UNITED STATES

## Abstract

Much work has been dedicated to identifying members of the microbial gut community that have potential to augment the growth rate of agricultural animals including chickens. Here, we assessed any correlations between the fecal microbiome, a proxy for the gut microbiome, and feed efficiency or weight gain at the pedigree chicken level, the highest tier of the production process. Because selective breeding is conducted at the pedigree level, our aim was to determine if microbiome profiles could be used to predict feed conversion or weight gain in order to improve selective breeding. Using 16s rRNA amplicon sequencing, we profiled the microbiomes of high and low weight gain (WG) birds and good and poor feed efficient (FE) birds in two pedigree lineages of broiler chickens. We also aimed to understand the dynamics of the microbiome with respect to maturation. A time series experiment was conducted, where fecal samples of chickens were collected at 6 points of the rearing process and the microbiome of these samples profiled. We identified OTUs differences at different taxonomic levels in the fecal community between high and low performing birds within each genetic line, indicating a specificity of the microbial community profiles correlated to performance factors. Using machine-learning methods, we built a classification model that could predict feed conversion performance from the fecal microbial community. With respect to maturation, we found that the fecal microbiome is dynamic in early life but stabilizes after 3 weeks of age independent of lineage. Our results indicate that the fecal microbiome profile can be used to predict feed conversion, but not weight gain in these pedigree lines. From the time series experiments, it appears that these predictions can be evaluated as early as 20 days of age. Our data also indicates that there is a genetic factor for the microbiome profile.

## Introduction

The lumen of the intestinal tract is inhabited by millions of microorganisms. This community, called the gut microbiome, is dominated by bacteria, some of which promote host health and nutritional benefits [1]. Fermentation by microorganisms in the gut can provide secondary metabolites and nutrients that can be absorbed by host intestinal cells. These metabolites include fatty acids (FA) such as butyrate, acetate, and propionate that are used as nutrients for host growth and health. Thus, the microbiome of the intestinal tract has a substantial impact on nutrient availability, which in turn, impacts the animal growth potential.

Identifying correlations between the microbiome, body weight, and nutrition is of great interest to human health, but agriculture can also apply this concept to the production of food animals. Reducing the amount of time, it takes to grow an animal to harvest weight and improving the feed efficiency of the animal is advantageous in terms of cost to the producer and consumer. With respect to chickens, selective breeding has greatly reduced growing time and improved feed efficiency [2]. These improvements are due primarily to selecting and propagating birds that have low feed conversion ratios and rapid weight gain. This selective breeding process is conducted at the top tier of a multi-level process [2]. The top tier is composed of the genetically well-defined pedigree chickens. The offspring of these chickens may be graded for performance metrics including weight gain and feed conversion as part of the selective breeding process. A very small portion of these offspring that have the desired metrics are chosen to remain in the pedigree tier. The others are used to produce production breeders, which composes the next tier. The bottom tier is composed of offspring of the production breeders, referred to as production birds. The production birds are chickens that are harvested, processed and sold for consumption.

Correlations between microbiome profiles and industry metrics of growth have been previously described in production level chicken lines [3,4,5,6,7,8,9]. Within these studies, bacterial operational taxonomic units (OTUs) within various taxonomic groups were identified and correlated with feed conversion ratio (FCR) and weight gain. These OTUs, that were associated with feed conversion ratio were distinctly different OTUs from those correlated with promotion or inhibition of growth [4,5,6]. However, as far as we know, correlations between performance metrics and the gut microbiome have not been investigated at the pedigree level. Additionally, the abundance dynamics of bacterial taxa correlated with high performance over a time series of host development has not been reported in pedigree chickens. Understanding how the fecal microbial community, a common proxy for the gut microbial community, varies at the level of pedigree lineages, and throughout host development has potential to improve production costs as well as future work seeking to manipulate the chicken gut microbiome [3,5,6,8]. Ultimately, being able to accurately predict correlations between production metrics and the microbial profile in pedigree lines could be used to improve the selective breeding process.

In this study, two distinct genetic lineages of pedigree breeders were used as previous studies have suggested that the gut microbiome varies across lineages of production chickens [4,5,6,8]. Performance measurements of chickens were designated as high or low weight and feed convertors (numerically defined in materials and methods section below). We used 16S rRNA amplicon sequencing to profile the fecal microbiome of high and low performing birds in two pedigree lines of chickens. Our aim was to determine if microbiome profiles could be used to predict common industry performance metrics. We also conducted a time-series to investigate how OTUs predictive of such performance and production measurements vary throughout the rearing process.

We used machine-learning methods to build a classification model that can predict feed conversion performance from the fecal microbial community. Our results suggest that the microbiome profile of high and low performing chickens, and the OTUs predictive of that performance, can differ between pedigree lineages. We found that the fecal microbiome of maturing chickens is dynamic during early developmental stages and more stable after 3 weeks of age. We also found that OTUs predictive of high feed conversion performance are differentially represented in the feces of young chickens.

## Materials and methods

### Ethics statement

All poultry were raised on Cobb-Vantress, Inc .farms under company specific protocols. Animal welfare standards met or exceeded those put forth by the National Chicken Council (NCC). All sampling was conducted by Cobb-Vantress employees. Academic institutions received only fecal samples. No animals were kept or raised in any of the academic institutions involved in this study and therefore, no IACUC was required for the study. No animals were sacrificed for this study.

### Poultry housing and sampling

Two lineages, phenotypically distinct of Cobb-Vantress breeders were chosen and labeled Line A and Line B. Specifically, for mature birds, the average body mass for Line A was 2150 g, and 3500 g for Line B. All birds were vaccinated for common viral and bacterial diseases following manufacturer’s protocols. A total of 3 independent experiments were conducted in which the microbiome composition was evaluated: 1) a maturation time series; 2) feed conversion performance; and 3) weight gain performance. For each experiment a separate group of 200 chickens was used. Lines A and B birds for each experiment were housed separately.

In the maturation time-series, all chickens were wing tagged so that the same chicken could be sampled consecutively throughout the experiments. Line A was sampled at day of hatch, then again at 2, 3, 5, 6 and 16 weeks of age. Line B was sampled at day of hatch, then again at 2, 3, 6, 9, and 16 weeks of age. Timing of fecal sample collection were concurrent with changes in feed formulation. Formulation of the feed, corresponding to common industrial rearing protocols of decreasing dietary protein over time, occurred prior to fecal sample collection at each time point. The time points covered a developmental range from egg hatching, performance grading, to breeder selection. A total of 576 samples were collected, 48 of which were sequenced reflecting 4 samples at 6 different ages for both lines used.

In the performance grading experiments, Line A and B birds were graded for weight gain, defined as the weight gained by an individual bird over time; and feed conversion, a calculation of feed consumed divided by weight to measure the efficiency of feed conversion to body mass. Within each line, birds were sorted into high and low performing groups. All four groups of chickens, designated by lineage and performance, were housed in separate pens. For Line A chickens, weight gain and feed conversion were measured at 5 and 6 weeks of age, respectively. For Line B, weights and feed conversion were measured at 6 and 9 weeks of age, respectively. Differences in grading and sample collection age are consistent with industry protocols for the two genetic lines used in this study. A total of 20 samples from each line were collected for each of the grading metric experiments, after the weights and feed conversion data were collected. Specifically, 10 samples from high and 10 samples from low performing chickens for both performance metrics. For Line A, high and low weight gain was defined as 2403.5±166.81g and 1876.0±249.47g of body mass, respectively, and high and low feed conversion performance was defined as 1.45±0.90 and 1.71±0.21, respectively. For Line B, high and low weight gain was defined as 3928.5±373.6 and 3009.0±385.6g of body mass, respectively, and high and low feed conversion performance was defined as 1.77±0.09 and 2.36±0.13, respectively. Because numerically lower feed conversion scores reflect desirable performance, a bird with a lower feed conversion score would be categorized as higher performing than a bird with a higher feed conversion score. Fecal samples and weight data were collected from males and females in the maturation time series and in the weight gain grading experiments. However, in the group of birds graded for feed conversion, fecal samples and feed conversion data were collected only from females.

All fecal samples were collected by placing sterile baking paper under individual chickens. On occasion, chickens were gently squeezed to expel feces onto the baking paper. Once a fresh fecal sample was available, it was placed into a sterile 50 mL centrifuge tube and stored on ice. Samples were frozen at -20°C until the DNA could be extracted. Fecal samples were utilized in this study due to concerns that cloacal swabs may not collect enough material for sufficient analysis and the need to not sacrifice birds in the maturation time series so that individuals could be tracked over time.

### DNA extraction, amplicon preparation, and sequencing

The DNA from all fecal samples was extracted in an individual manner. Fecal samples were weighed (100 mg) and DNA was extracted using a bead-beating procedure. DNA extracted was processed with the QIAmpDNA Stool Mini Kit (Qiagen, Valencia, CA) according to the manufacturer´s guidelines to remove PCR fecal inhibitors. Extracted DNA was quantified by spectrophotometry using a Qubit fluorometer (Life Technologies, Grand Island, NY) prior to storage at -80°C.

The V4 hypervariable region of the 16S rRNA gene was amplified from extracted DNA using primers 515F (GTGCCAGCMGCCGCGGTAA) and 806R (GGACTACHVGGGTWTCTAAT) as previously described by Caporaso et al. [10]. All PCR reactions were performed using 25 μL containing 1x Five Primer Hot Master Mix (5 PRIME, Fisher Scientific Company,LLC, Pittsburgh, PA) and 0.2 μM of each primer, PCR grade water (MoBio Laboratories, Inc., Carlsbad, CA) and 50 ng DNA template. The PCR conditions used were 94°C for 3 min; 35 cycles of 94°C for 45 sec, 50°C 60 sec and 72°C 90 sec; followed by 72°C 10 min. Three amplification reactions were conducted for each sample (25 μL per PCR reaction). After amplification, the PCR product was evaluated on a 1.5% agarose gel to confirm the target band size of 350 bp. Amplicon quality and concentrations were measured using a QuBit fluorometer (Life Technologies). Samples were then combined in equal concentrations (240 ng/sample) and cleaned using the MoBio UltraClean PCR Clean Up Kit (MoBio Laboratories, Inc., Carlsbad, CA) following the manufacturer’s instructions. Samples were checked for quality and concentration using the Bioanalyzer 2100 and Agilent High Sensitivity DNA kit (Agilent Technologies, Palo Alto, CA, USA). All cluster generation and paired-end sequencing for 300 bp read length was done on the Illumina next generation sequencing MiSeq system using the Illumina MiSeq V2 chemistry following the manufacturer´s protocols.

### Sequencing data processing and OTU analysis

From the sequencing data, forward and reverse reads were merged and filtered by estimated error per read using USEARCH (version 8) (see supplemental methods). After error filtering, the data further processing and clustering of OTUs was performed using an in-house pipeline (see Supplemental Methods). OTUs were taxonomically classified using the Ribosomal Database Project (RDP) with a confidence cut off of 0.5 [11].

Samples with less than 500 total reads and OTUs present in less than a total of three subjects were filtered out using custom python scripts (Python version 3.5.2) and were not considered in down-stream analysis. A total of 1,025 OTUs and 121 samples were retained and included in downstream analysis. All analyses were performed on an OTU table of normalized fractions generated by dividing the count of each OTU by the total number of reads present for a sample. Natural log was used for all log transformations of the normalized OTU abundance data. Log transformation of the normalized OTU table was performed by adding a scalar to all values in the table. The scalar used, -13.77, was calculated as one half multiplied by one divided by the maximum total sample read count in the un-normalized OTU table. Alpha (Shannon diversity) and beta (Jensen-Shannon divergence) diversities [12] were calculated using Qiime [13] and pysurvey, respectively (See supplemental methods).

### Identification of performance differentiating features in the microbiome

To measure and quantify differences between groups classified as high and low performance in weight gain or feed conversion, Linear Discriminant Analysis (LDA) was performed using LEfSe (Linear Discriminant Analysis Effect Size) and following steps and defaults recommended by the authors (https://huttenhower.sph.harvard.edu/galaxy/) [14]. Taxonomic group comparisons were conducted at the Phyla, Class, Order and Family levels. Analyses were preformed separately for each experiment and genetic line.

Normalized OTUs with an average relative abundance greater than 1x10^-6^ were subjected to univariate analysis. Specifically, a Mann-Whitney U-test (scipy package in Python) was conducted between individual OTU relative abundances in high and low performing groups. P-values calculated in significance testing were then corrected for false discovery rate (FDR) using a the Benjamini-Hochberg FDR method (statsmodels package in Python) to generate q-values. OTUs that were identified as discriminating using LDA were then filtered for those with q-values greater than 0.2.

### Identification of performance predictive features in the microbiome

Correlations with metadata (age, weight, feed conversion) treatment were conducted using a random forest machine learning approach [15]. All OTU abundance data were analyzed with a log transformation. All metadata categories were analyzed without transformation. All parameters utilized can be found in supplemental methods. Model accuracy was determined by calculating the area under the curve (AUC) of the corresponding receiver operating characteristic curve. Model accuracy was also determined by calculating the significance of the true and predicted values of the corresponding confusion matrix using a Fischer´s Exact Test. Top predictive OTUs contributing to the models were ranked using mean decrease accuracy (MDA). Correlations of OTU abundances and continuous metadata were determined using Kendall-Tau correlation coefficients.

Phylogenetic trees were produced for organization and visualization purposes using unique OTU representative sequences. Prior to building a phylogenetic tree, QIIME was used to align OTU unique sequences to full-length 16S reference sequences in the greengenes database (version gg_13_8) [16]. The aligned sequences were trimmed of gaps using trimAL (version 1.2) [17] and a constraint file created from the RDP taxonomic classifications was generated using a custom Python script. FastTree [18] was then used to build the phylogenetic tree (see Supplemental Methods). Abundance heat maps associated with the phylogenetic trees were generated using the Interactive Tree of Life tool [19].

## Results and discussion

### The microbiome is dynamic and changes during host development

**Fig 1:**
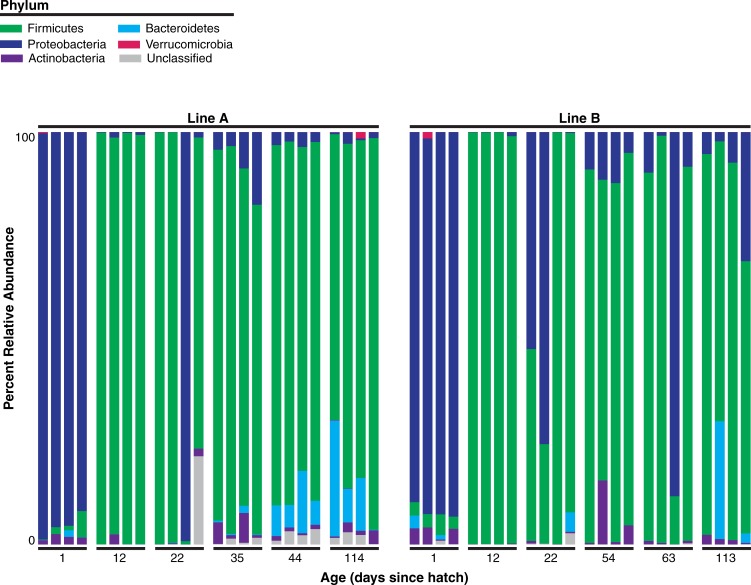
The microbiome is dynamic and differs between genetic lines during host development. Bird age is indicated in weeks after hatch.

Shifts in the microbial community, observed as relative abundances at the phyla level, occurred throughout the rearing process (Fig 1). At hatching, the fecal microbial communities of both lines are dominated by the phylum *Proteobacteria*. Over the course of development, both lines show a switch to a community dominated by the phylum *Firmicutes* as well as increasing abundances of the phyla *Actinobacteria*, and *Bacteroidetes*, as previous studies described [20,21]. These studies in chickens, as well as other animals, report that the microbiome is typically dynamic and populations change with the histological development and environmental conditions [20,21,22,23,24]. It is possible that dietary changes that occurred throughout the rearing process, could also affect the microbial community and contribute to the community dynamics observed in this study.

While phylum-level trends were similar between Lines A and B, there are notable distinctions. Specifically, the two lines differed in the abundances of phylum *Bacteroidetes* in later developmental stages (Fig 1). In Line A chickens, Bacteroidetes are consistently abundant at 6 and 16 weeks of age. In Line B chickens, only one chicken had a substantial abundance of Bacteroidetes at 16 weeks of age. These differences in phylum abundance between the genetic lines may be partly due to genetic effects. It is also possible that “pen” biases could be contributing to these trends as the two lines were housed in separate facilities. Additionally, the differences in ages between the two lines during the 4^th^ and 5^th^ sampling time points may contribute thus limiting the comparisons that can be made at those time points.

### A majority of OTUs in the gut microbiome persist over host development

**Fig 2:**
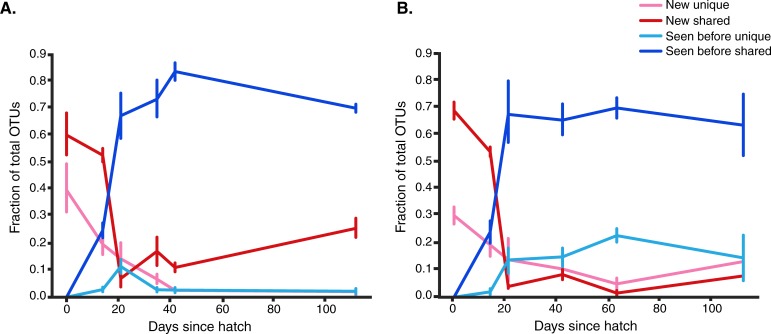
During host development a majority of the OTUs are persistent and not unique. OTUs are defined as “Seen before” if they were observed in at least one previous time point, and “New” if they have never been observed in a previous time point. OTUs are defined as “unique” if they have been observed in only one chicken, and “shared” if they are observed in at least 2 chickens. Trends in OTU persistence as shown for Line A **(A)** and Line B **(B)** chickens.

During early phases of growth, primary colonization and increasing diversity was followed by a community that was dominated by OTUs that persist between time points. Additionally, these persisting OTUs are common and are not unique to an individual chicken, suggesting increasing community stability over-time. Specifically, between hatch and 2 weeks of age, occurrences of OTUs that were not observed in previous time points were more likely to be shared between at least two chickens, rather than being unique to a single chicken (Fig 2). While new OTUs were observed throughout the time series, at 3 weeks of age the fraction of previously observed OTUs, that were observed in at least two different birds, was greater than 65% for both genetic lines. Additionally, after 3 weeks, less than 20% of the total observed OTUs were unique to a single bird. From these observations we can infer that after 3 weeks of age the chicken fecal microbiome became highly stable and consistent across chickens of the same genetic lineage. A similar pattern has been reported in the successional dynamics and subsequent community stability in the gut microbiome during the course of human and mouse developmental maturation [22,23,24,25].

### Phylum level differences between high and low grading differ by genetic line and grading metric

**Fig 3:**
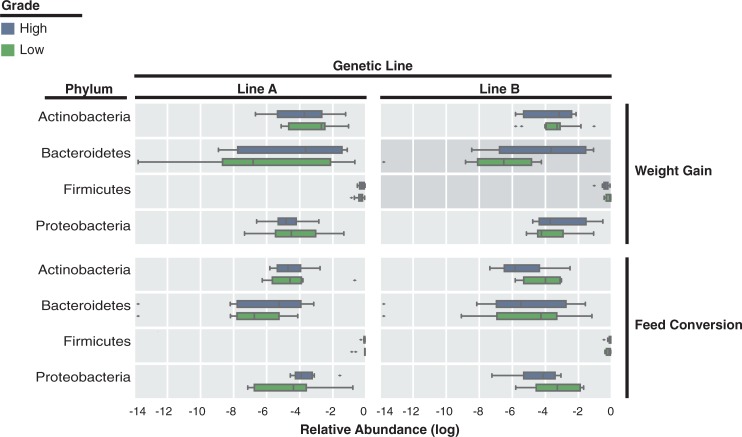
There are phylum level differences between genetic lines and high or low performance graded adult chickens. We illustrate the log relative abundances of phyla observed in Line A and B chickens graded using the weight gain and feed conversion metrics. Only phyla with a non-log transformed mean abundance of at least 1% are included. Phyla that significantly differ between high and low chickens are indicated with a darker tinted background. Significance is defined as a p-value less than or equal to 0.05 using an uncorrected Mann-Whitney U-test.

A comparison between high and low performing chickens revealed differences in phyla level abundances comparing both genetic lines and grading metrics (Fig 3). In Line A chickens, there were no significant differences between high and low performing chickens at the phylum level for either metric. However, a single OTU classified as *Chloroplast* has a significantly higher abundance in low performing chickens using both the weight gain and feed conversion metrics (S2 Fig). *Chloroplast* reads are often observed in human and animal gut 16S rRNA studies. It has been suggested that these reads are derived from off-target amplification of food related plant material and are often removed prior to downstream analysis [26,27,28]. However, there are studies where the abundance of *Chloroplast* reads have been utilized as a proxy for abundance of plant dietary content [29,30] or low bacterial biomass [31]. The significantly higher abundance of *Chloroplast* in poorly performing Line A chickens could indicate a higher consumption of feed with low increases in body mass or an overall lower bacterial biomass in the guts of these chickens.

Line B chickens differed significantly in the abundance of the phyla *Bacteroidetes* and *Firmicutes*, between high and low weight gain graded chickens (Fig 3). Line B chickens graded as high or low using feed conversion, also differed moderately in the abundance of *Firmicute*s, although this difference was only marginally significant (p = 0.05). Similarly, no correlation between weight and microbial composition has been reported in production level chickens [9]. A previous study reported a significant increase in the relative abundance of *Firmicutes* in poor performing chickens [5]. These conflicting results among studies may reflect the effects of environment and genetic background on the gut microbiome of chickens.

Given the phylum-level differences observed between genetic lines and grading metrics, it is important to note that with the exception of Line B chickens graded using the weight gain metric, there are no significant differences in alpha diversity between high and low graded chickens (Fig 3). This may indicate that weight and feed conversion performance are dictated by specific bacterial groups in the community and are not a function of diversity as indicated in some human weight gain and obesity studies [32, 33].

### OTUs discriminating between high and low performance are specific for genetic line and grading metric

**Fig 4:**
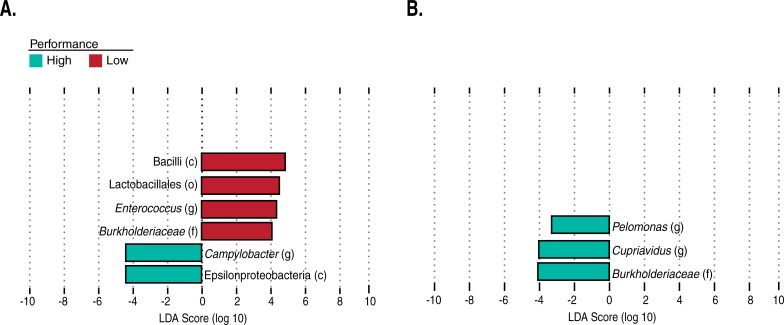
LDA and univariate analysis identify taxa that discriminate between high and low performing chickens. Histograms of the LDA scores show differentially abundant features in high (blue) and low (red) **(A)** Line B chickens graded with weight gain; and **(B)** Line A chickens graded with the feed conversion metric. Only taxonomic groups representing features identified as discriminating in LDA analysis that also had a q-value less than or equal to 0.2 are included. Taxonomic levels are displayed as c, o, f, and g indicating class, order, family, and genus levels respectively.

Using LDA analysis, we identified features in the fecal microbiome that significantly differed in abundance between high and low performing Line B birds graded by weight gain and Line A birds graded by feed conversion (Fig 4). When comparing high and low performing weight gain groups in Line B birds, OTUs belonging to the clade *Campylobacteraceae* were enriched in the high performing group (Fig 4A). In the chicken intestinal tract, *Campylobacter* is a commensal that typically colonizes the chicken intestinal tract around 2 and 3 weeks of age [33,34]. *Campylobacter* is thought to promote the production of short chain fatty acids (SCFAs) by serving as a hydrogen sink [34,35]. For this reason, higher concentrations of these bacteria could lead to an increased production of organic acids that can be used as an energy source for the host [36]. It is important to note that due to the use of V4 16S rRNA amplicon analysis in this study we are limited to the genus level of taxonomic resolution. The genus *Campylobacter* contains non-pathogenic and pathogenic organisms and pathogenesis cannot be determined from genus level 16S rRNA gene classification alone.

Low weight gain performing Line A chickens were enriched for many OTUs in the phylum *Firmicutes*. Specifically, OTUs classified as *Bacilli*, *Lactobacillales*, *Burkholderiaceae*, and *Enterococcaceae* (Fig 4A). There are confounding reports on the beneficial effects of these bacteria on nutrient availability and host body weight. The order *Lactobacillales*, in particular, were associated with poor weight gain and the production of bile salt hydrolase (BSH) in poultry and pig models [37,38,39,40]. Yet, *Lactobacilli* are widely used in commercial probiotics for production level chickens and have been reported to increase weight gain [41]. However, it is worth noting that the improved weight gain reported in these studies is strain-specific and typically occurs during early stages of growth [42].

OTUs classified as *Pelomonas*, *Cupriavidus*, and *Burkholderiaceae* were enriched in high feed converting birds when comparing high and low feed conversion groups in Line B (Fig 4B). We maintained these OTUs in our findings, because they were also identified as predictive features in our classification model described below. The identification of the genus *Cupriavidus* as being enriched in high feed converting chickens could actually be derived from feed and not the gut community, similar to *Chloroplast* as described above.

It is important to note that LDA also identified discriminating OTUs in the cases of Line A weight gain and Line B feed conversion chickens. However, none of those identified OTUs were significant after FDR correction.

### Microbiota composition predicts feed conversion performance

**Fig 5:**
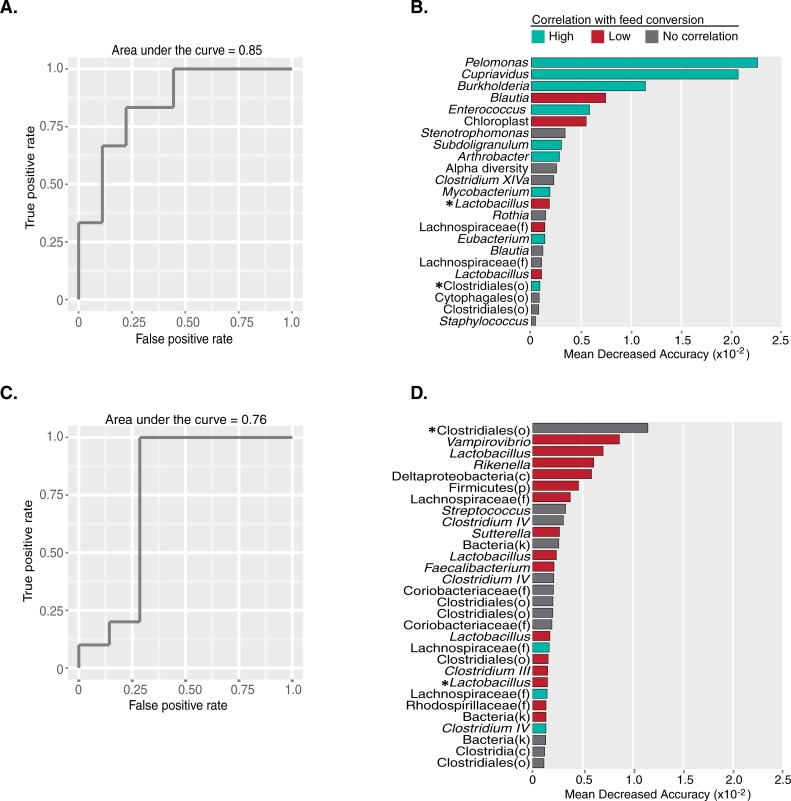
Microbiota composition predicts feed conversion grading. This figure depicts the accuracy, as determined by AUC analysis of ROC curves, of the models built to predict high verses low feed conversion performance from the microbiome composition of Line A and B chickens (**A, C**, respectively). The top contributing OTU features of those models are shown for Line A and B in **B and D**, respectively. Taxonomic level for each OTU is displayed is genus unless otherwise noted. Phyla, class, order, and family levels are indicated as p, c, o, and f, respectively. Correlations of OTU abundance and continuous feed conversion scores, calculated using Kendall-Tau correlation, are indicated as high (teal, Tau coefficient < -0.3), low (red, Tau coefficient > 0.3), and no correlation (grey, Tau coefficient < absolute value of 0.3). Because numerically lower feed conversion scores reflect desirable performance, an OTU with a negative correlation was deemed as contributing to high performance and an OTU with a positive correlation was deemed as contributing to low performance. * Indicates OTUs shared between **B and C**.

Given the distinctions in microbial composition between high and low performing birds observed at the phylum level and in the LDA analyses, we asked whether these differences were robust enough to enable the classification of performance from the fecal microbial community within a genetic line and grading method. We built a random forest classification model based on the microbiota composition in order to predict high or low weight gain or feed conversion performance within the two pedigree genetic lines. Using this model, we were able to accurately predict if chickens were from high or low feed conversion groups in Lines A and B, with AUCs equal to 0.85 (p = 0.04, Fisher´s Exact test) and 0.76 (p = 0.003, Fisher´s Exact test), respectively (Fig 5A and 5C, S1 Table).

The classification models built for the prediction of high or low weight gain performance were less effective than those for feed conversion. For Line A chickens, the classification was less accurate than that for feed conversion, AUC = 0.67, and the predictive power was insignificant (p = 0.17) (S1 Table). The classification model built for Line B chickens was equally accurate to that of feed conversion, AUC = 0.76, but the predictive power was insignificant (p = 0.17) (S1 Table).

The finding that the feed conversion classifier was more accurate than the weight gain classifier is consistent with a history of difficulties in predicting weight gain and obesity from gut microbial composition [43]. Nonetheless, there is substantial evidence connecting the gut microbiota and nutrient availability which would be reflected in the feed conversion data as nutrient intake and gain in body mass are used to calculate FCR [35, 40, 44, 45,46].

### OTUs that predict performance are distinct to genetic lines

OTUs contributing to the predictive models of feed conversion performance differed between the two genetic lines. In the feed conversion classifiers, only two OTUs, family *Clostridiales* and genus *Lactobacillus*, of the top predictive OTUs were common in both Line A and Line B models (Fig 5B and 5D). For Line A chickens, a majority of the model predictive OTUs had abundances that correlated with favorable feed conversion performance (Fig 5B). For Line B chickens, all of the predictive OTUs, with the exception of one OTU identified as *Clostridium IV* and two as family *Lachnospiraceae*, correlate with poor feed conversion performance or no correlation at all (Fig 5D). It is interesting that OTUs classified as *Lactobacillus*, *Lachnospiraceae*, and *Clostridiales* are highly predictive of feed conversion in both genetic lines (Fig 5B and 5D). These results are consistent with previous studies that have indicated a correlation between *Clostridia*, *Lactobacillus*, and *Lachnospiraceae* and improved or diminished performance grading [4,5,47].

As mentioned above, our LDA analysis comparing OTU abundances between favorable and poor feed conversion performing chickens resulted in only two OTUs maintaining a q-value less than or equal to 0.2 for both Lines A and B. This indicates that while each of the OTUs identified in our model do not individually have a strong correlation between abundance and feed conversion to be considered significantly different across groups, as a community they are predictive of feed conversion performance.

### OTUs that predict performance are dynamic across a maturation time series

**Fig 6:**
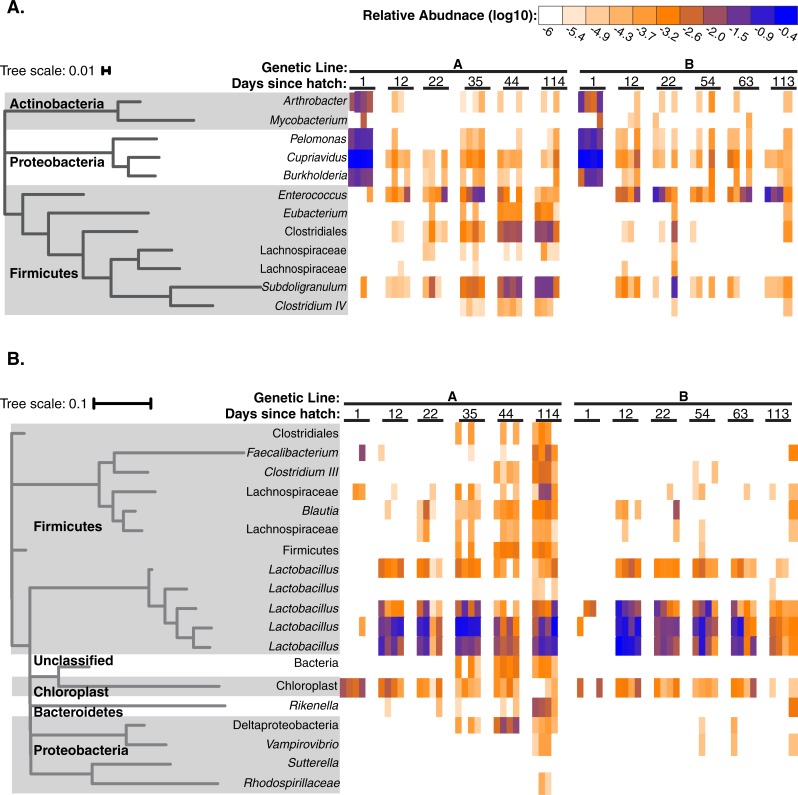
Top predictive OTUs correlated feed conversion performance are dynamic during host development. Relative abundance of top predictive OTUs that correlate with high performance (**A**) and low performance (**B**) are shown across a maturation time series in Line A and B chickens. Bird age is indicated as weeks since hatch.

To investigate the presence and abundance of OTUs that were predictive of feed conversion performance in developing chickens, we assessed the relative abundances of these OTUs within a maturation time series (Fig 6). An interesting trend appeared within the phyla *Proteobacteria* and *Firmicutes* that was independent of the chicken lineage. In birds with favorable FCR, the pattern followed a typical description of microbiome dynamics that has been described in chickens and other animals as well as humans [4,5,48]. In this pattern, the *Proteobacteria* were dominant at hatch, but were reduced after 2 weeks of age, at which time the *Firmicutes* began to appear and steadily increased in abundance over the time series. Conversely, in poor performing chickens *Proteobacteria* OTUs were virtually absent until 5 weeks of age and beyond (Fig 6B). In all groups the *Firmicutes* appear at 2 weeks of age. However, the poor FCR groups were dominated by genus *Lactobacillus* through the time series, while in the favorable FCR groups, *Lactobacilli* were absent. These differences in the patterns of Proteobacteria colonization and the Firmicutes taxonomic groups are a striking finding. Specifically, it is intriguing that the OTUs predictive of the favorable FCR groups followed a colonization pattern frequently described in chickens, humans and other animals consisting of early colonization by Proteobacteria followed by domination of the Firmicutes. Moreover, the association of *Lactobacillus* with poor feed conversion over a time series further questions the practice of using *Lactobacillus* as probiotics by the poultry industry [4, 41,42].

In addition to the colonization pattern of the Firmicutes, we found nearly half the Firmicutes associated with poor feed conversion were lactic acid producers. Lactic acid is not used as a nutrient for host cells. However, can be beneficial by lowering the pH, which in turn, can reduce the risk of colonization by zoonotic bacteria [49].

In contrast with poor FCR, all the OTUs in the Phylum Firmicutes that predicted favorable feed conversion were producers of the short chain fatty acids (SCFA). It should be noted that the bacterial taxa that ferment carbohydrates to produce specific SCFA are polyphyletic and some have the ability to produce more than one FA [50,51]. Nevertheless, many of the OTUs correlated with desired feed conversion belong to bacterial groups known to contain butyrate-producing species [7,52]. Colonic cells use butyrate as an energy source, but also have anti-inflammatory effects, encourage epithelial cell proliferation, can impact weight gain and has been correlated with desired performance in chickens [3,7,46,52,53].

In poor FCR birds, half of Firmicutes OTUs were *Lactobacillus* which are lactic acid producers (Fig 6B). In the remaining OTUs, other taxa including, *Faecalibacterium* were correlated with poor feed conversion. This genus is commonly considered beneficial as the majority are butyrate producers [51, 54]. However, functional differences at the species or strain level might have different effects as well.

Other OTUs correlated with poor feed conversion have been identified as acetate and propionate producers in other studies. Specifically, OTUs classified as *Blautia spp*., as well as some *Lachnospiracea spp*. (Fig 6B) [7,40, 54,53]. Previous studies have found evidence for high levels of propionate and acetate production in the chicken gut [7,35]. Propionate can contribute to weight control and is thought to do so through the enhancement of satiety [52,53]. Acetate production has been linked to weight control independent of satiety [51,52].

In light of the possible correlation between propionate and acetate production and undesirable feed conversion phenotypes, it is important to keep in mind that both propionate and acetate play important roles in healthy gut metabolism. Propionate and acetate serve important roles in the liver and other peripheral tissues [46]. High acetate concentrations have been shown to favor the growth of beneficial bacterial communities, including *Bifidobacterium* [55]. Additionally, acetogenesis can play an important role in the gut community by providing a hydrogen sink and serving as a potential precursor for the synthesis of butyrate [46,50]. The complexity of nutritional and metabolic relationships in gut communities introduces many challenges in elucidating microbial-host interactions and the reliability of predicting host phenotype from microbial composition, let alone the targeted manipulation of the gut community to develop the desired phenotype.

If successful colonization of young chickens is required to reliably select for the gut community of a desired phenotype, then differences in the abundances of butyrate producing bacteria during the maturation time series could help inform future work. For example, the OTUs classified as the genera *Enterococcus*, *Subdoligranulum*, *Pelomonas*, *Cupriavidus*, *and Burkholderia* (Fig 6A) are present in at least one favorable FCR chicken during hatch and persist through out the time series. This indicates that they are naturally present in young chickens and therefore may be candidate markers for early selective breeding. In contrast, the OTUs classified as genus *Eubacterium* and family *Lachnospiraceae* (Fig 6A) are absent or very sparse in young chickens and may not be useful as a predictor of FCR for selection.

## Conclusions

In this work, we have identified bacterial groups that are predictive of feed conversion in breeder pedigree lines, which represent the highest level of selection in the commercial poultry industry. We have identified OTUs that are predictive of desired feed conversion performance that are present during early life stages and persist into adulthood. These results reflect great potential for utilizing fecal microbial composition as an indication of future performance grading. Through sequencing and rigorous analysis of fecal samples, the identification and persistence of particular taxonomic groups could enhance traditional performance grading processes. Additionally, our work suggests that including maturation time series data may allow for more informed selection of candidates for future probiotic work. Alternatively, manipulation of the gut microbiota early in life could be used to encourage desired traits independent of genetic lineage.

## Supporting information

S1 Table Classification model statistics. (DOCX)

S1 Fig Chloroplast associated reads differ significantly between high and low graded Line A chickens. A darker tinted background indicates a significant difference within that genetic line between high and low chickens. Significance is defined as a p-value less than or equal to 0.05 using a Mann-Whitney U-test.
(EPS)

S2 Fig Shannon Diversity does not significantly differ between high and low graded chickens in most cases. In this figure we present the alpha diversity (calculated as Shannon diversity) for high and low graded Line A (A) and Line B (B) chickens; as well as Line A (C) and Line B (D) chickens graded using the feed conversion metric. Significance is defined as a p-value less than or equal to 0.05 using a Mann-Whitney U-test.
(EPS)

S1 Methods Supplementary methods used in this work. (DOCX)
