# Supplementary figures and images for: Using fecal microbiota as biomarkers for predictions of performance in the selective breeding process of pedigree broiler breeders
Source: PLoS One. 2019 May 7;14(5):e0216080. doi: 10.1371/journal.pone.0216080 (PMC6504170; doi:10.1371/journal.pone.0216080)

STable 1. Classification model statistics.
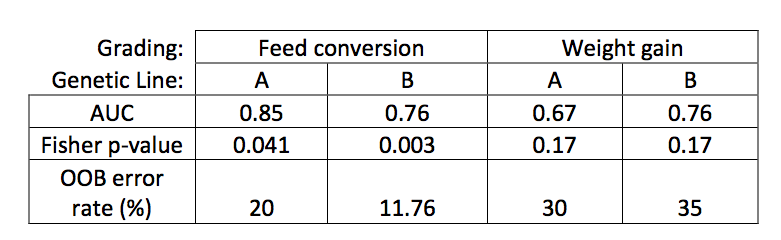

Supplement: S1 Table — (DOCX) [file pone.0216080.s001.docx]
