# Supplementary material for: Using fecal microbiota as biomarkers for predictions of performance in the selective breeding process of pedigree broiler breeders
Source: PLoS One. 2019 May 7;14(5):e0216080. doi: 10.1371/journal.pone.0216080 (PMC6504170; doi:10.1371/journal.pone.0216080)

**Supplemental Methods**

*Sequencing data processing and OTU analysis*

Forward and reverse read FASTQ files were merged and the estimated error per read was determined using the usearch (version 8) command –fastq_mergepairs with options -eetabbeout, –fastq_ascii 33 (Edgar, 2010). Merged reads were then filtered for an estimated error rate of 1 using the usearch8 command –fastq_filter (–fastq_maxee 1).

Further processing and clustering Operational Taxanomic Units (OTUs) was performed with an inhouse pipeline ( https://github.com/thomasgurry/amplicon_sequencing_pipeline). Within the pipeline, sequences of less than 250 bases were filtered out, 97% denovo OTUs were clustered using the UPARSE algorithm (Edgar, 2013) in usearch8 (Edgar, 2010), and OTUs with less than two reads were removed.

All custom python scripts were written using Python version 3.5.2 and can be located on github (https://github.com/arperrotta/public_code).

After initial processing a total of 11,036,040 sequence reads were generated with an average of 91,967 reads per sample (±112,735, n=120).

Shannon diversity was calculated using the command alpha_diversity.py with the metric option set to Shannon Diversity (-m shannon) (Caporaso, Kuczynski, et al. 2010). The Jensen-Shannon divergence was calculated using the dist_mat (metric=’JS’) command in pysurvey (https://bitbucket.org/yonatanf/pysurvey, https://github.com/swo/pysurvey).

*Identification of predictive features in the microbiome*

All OTU abundance data were analyzed with a log transformation on the OTU abundances by including the parameter --x_log. All metadata categories were analyzed without transformation. The analysis was run using three forests by including the parameter -n 3. The data was shuffled 100 times for p-value calculations by including the parameter -s 100. Class balancing was performed via downsampling using the parameter –b downsample. All other parameters were default.

*Description of tools and parameters utilized to generate phylogenetic trees.*

Aligned sequences used for tree building were trimmed of gaps using trimAL (version 1.2) with a gap threshold of 0.05 so gaps present in 95% or more of the sequences would be removed (-gt 0.05)(Capella-Gutiérrez, Silla-Martínez, and Gabaldón 2009). FastTree (Price, Dehal, and Arkin 2009) was then used to build a phylogenetic tree from the trimmed sequences using nucleotide alignment (-nt), a GTR+CAT model (-gtr), and constraint file created from RDP classifications described above (-constraint). The constraint file was generated using custom a Python script (Python 3.5.2).

LeFse analyisis

The non-parametric factorial Kruskal-Wallis sum rank test was used to detect overabundant taxa in each class. The Wilcoxon rank-sum test was then used to determine if the overabundant taxa were consistent among microbiomes within a group. Finally, the Linear Discriminant Analysis (LDA) was used to estimate the effect size of each overabundant taxa (13). For each individual sample the relative abundance of each taxa assignments was included to construct the LEfSe analysis matrix .Taxonomic group comparisons were conducted at the Phyla, Class, Order and Family levels. Analyses were preformed separately for each sub-project and genetic line.

**Supplementary Figures.**

SFigure 1. Chloroplast associated reads differ significantly between high and low graded Line A chickens. A darker tinted background indicates a significant difference within that genetic line between high and low chickens. Significance is defined as a p-value less than or equal to 0.05 using a Mann-Whitney U-test.

SFigure 2. Shannon Diversity does not significantly differ between high and low graded chickens in most cases. In this figure we present the alpha diversity (calculated as Shannon diversity) for high and low graded Line A (A) and Line B (B) chickens; as well as Line A (C) and Line B (D) chickens graded using the feed conversion metric. Significance is defined as a p-value less than or equal to 0.05 using a Mann-Whitney U-test.

STable 1. Classification model statistics


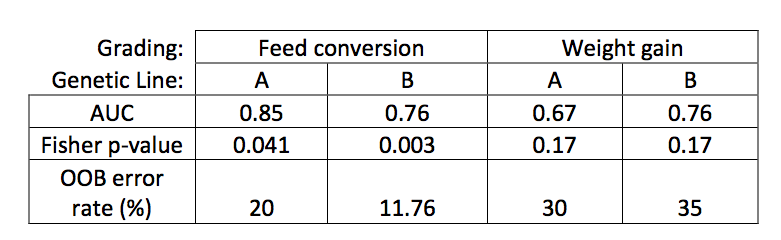

Supplement: S1 Methods — (DOCX) [file pone.0216080.s004.docx]
